# Clinical and immunological characterisation of patients with common variable immunodeficiency related immune thrombocytopenia

**DOI:** 10.1007/s10238-023-01166-2

**Published:** 2023-09-05

**Authors:** Nadia Somasundaram, Oliver Meyer, Carmen Scheibenbogen, Leif Gunnar Hanitsch, Anna Stittrich, Uwe Kölsch, Kirsten Wittke

**Affiliations:** 1 Charité - Universitätsmedizin Berlin, corporate member of Freie Universität Berlin and Humboldt-Universität zu Berlin, Institute of Medical Immunology, Augustenburger Platz 1, 13353 Berlin, Germany; 2 Red Cross Blood Service NSTOB, Eldagsener Straße 38, 31832 Springe, Germany; 3 Labor Berlin - Charité Vivantes GmbH, Sylter Str. 2, 13353 Berlin, Germany

**Keywords:** CVID, ITP, Autoimmunity, Primary immunodeficiency

## Abstract

Primary Immune thrombocytopenia (ITP) is an autoimmune disease. Secondary ITP occurs in patients with underlying diseases such as common variable immunodeficiency (CVID). CVID is one of the most common symptomatic primary immunodeficiencies in adults, characterised by infectious and non-infectious symptoms. Amongst CVID patients, ITP is the most frequent autoimmune manifestation. In this single-centre study, we performed a clinical and immunological characterisation of 20 patients with CVID-related ITP and 20 ITP patients without CVID to compare severity and remission rates. We found that patients with CVID-related ITP had a higher WHO Bleeding Scale at initial diagnosis yet showed higher remission rates and required less treatment. Patients with ITP needed up to seven therapy options and were often treated with second-line drug therapy, whilst only one CVID-related ITP patient required second-line drug therapy. Therefore, we show that the course of thrombocytopenia in patients with CVID-related ITP is milder. Furthermore, we show that soluble interleukin-2 receptor (sIL-2R, CD25) was higher in CVID-related ITP compared to ITP patients and could accurately classify patient cohorts with an Area Under the Receiver Operating Characteristic of 0.92. Whilst none of the ITP patients had a history of immunodeficiency, we found immunological abnormalities in 12 out of 18 patients. Therefore, we recommend screening ITP patients for CVID and other immunodeficiencies to detect immune abnormalities early, as we found patients with reduced immunoglobulin levels as well as severe lymphocytopenia in our ITP cohort.

**Supplementary Information:**

The online version contains supplementary material available at 10.1007/s10238-023-01166-2.

## Introduction

Primary Immune thrombocytopenia (ITP) is an acquired autoimmune disease characterised by increased destruction and impaired production of platelets leading to isolated thrombocytopenia (< 100/nL) and subsequently to an increased bleeding risk [1–3]. Around 67% of adult patients with acute ITP develop a chronic ITP [4].

The severity of bleeding varies, and many patients may present with either no symptoms, minimal bruising, or petechiae. However, ITP patients may also present with episodes of severe bleeding such as hypermenorrhoea or mucosal bleeding, and potentially gastrointestinal or intracranial haemorrhages. Primary ITP is a diagnosis of exclusion [1, 5]. Secondary ITP occurs in the course of other diseases such as infections (e.g. hepatitis C virus, human immunodeficiency virus) or common variable immunodeficiency (CVID) [6].

CVID is a primary immunodeficiency (PID) with a prevalence of about 2–4:100,000 [7–9]. Amongst primary immunodeficiencies, CVID is particularly often accompanied by autoimmunity and other non-infectious manifestations [9–12]. According to the diagnostic criteria of the European Society for Immunodeficiency (ESID), CVID is defined by a reduced level of immunoglobulin G (IgG) and a failure to respond to immunisation [10], as well as decreased concentrations of IgA ± IgM [13, 14]. This results in a broad range of symptoms, mainly bacterial infections of the respiratory tract, such as chronic sinusitis, recurrent bronchitis, and pneumonia. Still, similarly to ITP, CVID remains a diagnosis of exclusion [13–15]. CVID patients often experience a significant diagnostic delay from the onset of initial symptoms until they receive the diagnosis and therefore treatment, typically in the range of 4 to 6 years. This delay is a major cause for morbidity in these patients [7, 8, 16].

Symptoms often begin between the ages of 20 and 50 [10, 17], yet can start at any age although CVID cannot be diagnosed before the age of four [10, 18]. In addition to recurrent infections, autoimmunity occurs in up to 30% of CVID patients [10, 14, 17, 19], with autoimmune cytopenias developing in approximately 10–15% of CVID patients [8, 20]. In the largest study on autoimmune cytopenias in CVID, a frequency of 7.4% was shown for ITP [8].

Currently, an increase in CD21^low^ B cells is the best-known predictor of autoimmune cytopenias in CVID patients [14, 21]. In patients with both CVID and ITP, autoimmune cytopenias are often diagnosed years earlier than the immunodeficiency [14, 19, 22]. They have also been reported to have a later age of onset of their immunodeficiency than those CVID patients without ITP [23]. This presents an opportunity to screen for immunodeficiencies amongst ITP patients, to reduce diagnostic delay.

The aim of this study was to analyse and compare the clinical course, WHO Bleeding Scale and immunological statuses of patients diagnosed with ITP and patients with CVID-related ITP. Hereby, we aimed to identify key characteristics in these patient cohorts.

## Methods

### Patient enrolment and clinical data

This monocentric, combined retrospective, and prospective study was performed at the Immunodeficiency Outpatient Clinic and the Institute of Transfusion Medicine of the Charité Universitätsmedizin Berlin, Campus Virchow Klinikum (Berlin, Germany).

**Fig. 1:**
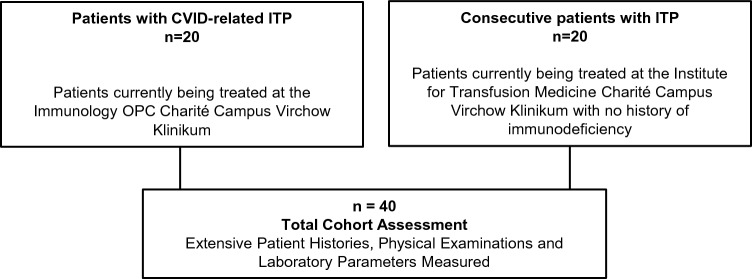
Patient Enrolment and Study Design

We enrolled all 20 patients diagnosed with CVID-related ITP from the outpatient clinic starting in May 2019. Differential diagnoses of thrombocytopenia in CVID-related ITP patients were extensively assessed by experts for immunodeficiency and a hematooncologist with expertise for ITP in order to rule out other causes for thrombocytopenia. Additionally, 20 patients with ITP were consecutively enrolled, as shown in Fig. 1. ITP was diagnosed in accordance with the German ITP Guideline (https://www.onkopedia.com/de/onkopedia/guidelines/immunthrombozytopenie-itp/@@guideline/html/index.html) [2]. At the time of inclusion in this study, none of the patients with ITP were suspected of having CVID or any other immunodeficiency. A later diagnosis of immunodeficiency after enrolment, however, did not lead to exclusion from the trial.

Simplified flow-chart of cohort design. Patients with CVID-related ITP were enrolled from the immunodeficiency outpatient clinic, whilst patients with ITP were consecutively enrolled from the institute for transfusion medicine. CVID = Common variable immunodeficiency; ITP = Immune thrombocytopenia; OPC = outpatient clinic.

CVID was diagnosed following the ESID criteria for CVID (https://esid.org/Education/Common-Variable-Immunodeficiency-CVI-diagnosis-criteria) [18] and classified using the EUROclass classification [23]. Patients who presented to the outpatient clinic whilst already under immunoglobulin therapy were re-evaluated, taking the effects of treatment on immunoglobulin levels into account.

### Retrospective clinical data collection

All available patient records from disease onset until the end of data collection on March 12, 2021 were reviewed for complete medical histories, treatments, laboratory and genetic results, and results of radiological or physical examinations.

### Laboratory data

Retrospective laboratory data were collected where available in the electronic medical record. From patients with incomplete retrospective laboratory data, further blood samples were collected for testing. All laboratory parameters were analysed in Labour Berlin Charité-Vivantes GmbH (Sylter Straße 2, 13,353 Berlin). Leucocytes were surface-stained and measured according to standard procedures. Briefly, whole blood was stained for CD3 (clone UCHT1), CD4 (clone SFCI12T4D11), CD8 (clone B9.11), CD16 (clone3G8), CD56 (clone N901), CD19 (clone J3-119), TCR α/β (clone IP26A), TCR γ/δ (clone IMMU510), CD45RA (clone J33), CD45R0 (clone UCHL1), and CD45 (J33) with monoclonal antibodies by Beckman Coulter and were measured analysed on a Navios-EX FACS (Beckman Coulter GmBH Krefeld Germany) Flow Cytometer.

### Genetic testing

We aimed to collect genetic data from all patients with CVID enrolled in the study. We used panel sequencing using a panel of immune genes and whole exome sequencing. DNA was extracted from the patient’s blood according to standard protocol. Target regions were enriched from ultrasound fragmented DNA (SureSelect Human All Exon V6, Agilent), and sequencing was performed as paired-end next generation sequencing (NGS) (Illumina, Inc. CA USA). Sequence data were analysed using our in-house NGS data analysis pipeline and aligned to the human reference genome (hg19). Variant prioritisation was based on allele frequency, variant type, location in the gene, bioinformatic prediction tools (MutationTaster, PolyPhen and CADD) [24–26], evidence from literature, classification in ClinVar [27] and, where applicable, segregation within the family.

### Statistical analysis

Statistical analysis was performed in R version 3.6.1 [28]. For comparisons of laboratory parameters between pairs of groups, we performed a Mann–Whitney U Test. For diagnostic accuracy, the Areas Under the Receiver Operating Characteristics (AUROC) with 95% confidence intervals using DeLong’s method [29] were calculated.

In tables, medians and interquartile ranges (IQR) are shown in all continuous data, and nominal variables are shown with frequencies and column percentages. For variables with missing data, the number of cases with valid data is shown.

## Results

### General characteristics

A cohort of 20 patients with CVID-related ITP, and 20 patients with ITP, was enrolled into the study, as shown in Fig. 1. Demographic and clinical characteristics of the total cohort are shown in Table 1. The median age at diagnosis for CVID amongst patients with CVID-related ITP was 35 years (interquartile range: 31.0 – 42.2) whereas they were diagnosed for ITP at 24.5 years (21.5–35.8). Patients with ITP tended to be older at initial diagnosis, with a median age of 37.5 years (25.5–46.0). The median retrospective observation period for the entire cohort was 15.7 years (9.1–19.0). This was defined as the period from which regular clinical and laboratory assessments were included in the analysis, starting from the initial diagnosis of either CVID or ITP until the end of the study in March 2021.

**Table 1:** Demographic and clinical characteristics at study inclusion

Characteristic	CVID-related ITP (*n* = 20)	ITP (*n* = 20)	*p-*Values
Retrospective observation period [years]	13.8 (9.7, 17.8)	15.8 (7.5, 19.3)	0.57
Age [years]	40.6 (38.2, 49.2)	47.4 (39.0, 63.1)	0.23
Sex [Female]	12 (60%)	13 (65%)	1.0
Age at CVID diagnosis [years]	35.0 (31.0, 42.2)	–	–
Age at ITP diagnosis [years]	24.5 (21.5, 35.8)	37.5 (25.5, 46.0)	0.10
Time from ITP to CVID diagnosis [years]	7.5 (− 0.5, 13.0)	–	–
Ig therapy at ITP diagnosis	3 (15%)	0 (0%)	0.23
AIHA	5 (25%)	4 (20%)	1.0
Accompanying autoimmunity^a^	7 (35%)	3 (15%)	0.27
Nodular lymphoid hyperplasia	4 (20%)	0 (0%)	0.11
Enteropathy	6 (30%)	0 (0%)	0.02
GLILD	5 (25%)	0/19 (0%)	0.05
Bronchiectasis	1 (5%)	1/19 (5%)	1.0
Granulomatous lesions	4 (20%)	0/19 (0%)	0.11
Splenomegaly	14 (70%)	2 (10%)	0.05
Splenectomy	5 (25%)	0 (0%)	< 0.01
Lymphadenopathy	12 (60%)	1 (5%)	< 0.01
Alopecia	1 (5%)	0 (0%)	1.0
Type B gastritis	2 (10%)	3 (15%)	1.0
Recurrent pneumonia	6 (30%)	0 (0%)	0.02
Recurrent sinusitis	13 (65%)	1 (5%)	< 0.01

Median and interquartile ranges (IQR) are shown for continuous variables, and frequency and column percentages for nominal variables. For variables with missing data, the number of valid cases is shown

*CVID* Common variable immunodeficiency, *ITP* immune thrombocytopenia, *IQR* interquartile range, *AIHA* autoimmune haemolytic anaemia, *GLILD* granulomatous-lymphocytic interstitial lung disease

^a^Autoimmune conditions as listed in supplemental Table S1

Fourteen of 20 (70%) patients with CVID-related ITP received the ITP diagnosis earlier than the CVID diagnosis, with a median delay of 7.5 years (-0.5 to 13.0). Sex was distributed similarly between both groups (CVID-related ITP: 60% female, ITP: 65% female).

### Course and therapy of ITP

Detailed characteristics, comparing patients with ITP and CVID-related ITP, are shown in Table 2. The WHO Bleeding Scale was used to classify the severity of bleeding symptoms at initial diagnosis. In our cohort, most patients exhibited only mild bleeding symptoms (WHO Grades 0–1). Patients with CVID-related ITP tended to have more frequently WHO Grade 1 at initial diagnosis than the patients with ITP (79 vs 50%).

**Table 2:** ITP clinical grading and treatment

	CVID-related ITP (n = 20)	ITP (n = 20)	*p-*Values
Remission	16/19 (84%)	0 (0%)	< 0.01
Relapses of those in remission	8/16 (50%)	–	–
Relapse before substitution dose IgG therapy	5/8 (62%)	–	–
*WHO bleeding scale*			
Grade 0	1/19 (5%)	9 (45%)	< 0.01
Grade 1	15/19 (79%)	10 (50%)	0.10
Grade 2	2/19 (11%)	1 (5%)	0.60
Grade 3	1/19 (5%)	0 (0%)	0.49
Grade 4	0/19 (0%)	0 (0%)	–
*Therapy lines*			
0	2 (10%)	2 (10%)	1.00
1	8 (40%)	0 (0%)	< 0.01
2	8 (40%)	5 (25%)	0.50
3	1 (5%)	4 (20%)	0.34
4	0 (0%)	2 (10%)	0.49
5	1 (5%)	4 (20%)	0.34
6	0 (0%)	1 (5%)	1.00
7	0 (0%)	2 (10%)	0.49
*Therapies*			
Corticosteroids	17 (85%)	18 (90%)	1.00
Immunoglobulins	10 (50%)	15 (75%)	0.19
Azathioprine	1 (5%)	5 (25%)	0.18
Rituximab	2 (10%)	2 (10%)	1.00
Sirolimus	1 (5%)	0 (0%)	1.00
Eltrombopag	1 (5%)	13 (65%)	< 0.01
Romiplostim	0 (0%)	7 (35%)	< 0.01
Anti D	0 (0%)	4 (20%)	0.11
Fostamatinib	0 (0%)	2 (10%)	0.49
No Therapy Ever	2 (10%)	2 (10%)	1.00

Median and interquartile ranges (IQR) are shown for continuous variables, and frequency and column percentages for nominal variables. For variables with missing data, the number of valid cases is shown. Remission is defined as no further episode of ITP for > 12 months, with > 100 platelets/nL and without treatment for the last eight weeks as defined by Grimaldi-Bensouda et al. for primary ITP (23)

*CVID* Common variable immunodeficiency, *ITP* immune thrombocytopenia, *OPC* outpatient clinic, *IQR* interquartile range

As described by Grimaldi-Bensouda et al. [30], remission of ITP is defined as a platelet count of > 100/nL, no further episode of ITP for more than 12 months and without specific treatment for the last eight weeks. Of the 20 patients with CVID-related ITP, 19 had sufficient laboratory and clinical assessments to apply the Grimaldi-Bensouda ITP remission criteria, including follow-ups over ≥ 12 months. Of these, 16 (84%) reached remission at least once during the study. Amongst these patients who experienced remission of ITP, 8 (50%) had at least one relapse during the observation period from beginning of their ITP until the end of the study. Of these 8 patients, only 3 (38%) received regular IgG substitution therapy at the time of the relapse. At the end of the study, all the included 19 patients with CVID-related ITP received regular immunoglobulin substitution-dose therapy. In comparison, none of the patients with ITP reached remission during the study observation period.

Patients with CVID-related ITP were less likely to require second- and further-line ITP drug therapies than patients with ITP. Most patients in both groups received corticosteroids for first-line treatment (CVID-related ITP: 17 (85%) vs. ITP: 18 (90%)). Second-line treatment was more frequent in patients with ITP and consisted mainly of Thrombopoietin receptor agonists (65% Eltrombopag, 35% Romiplostim). Only one patient with CVID-related ITP (5%) received Eltrombopag.

### Splenomegaly and autoimmunity

Splenomegaly was reported in 14 (70%) CVID-related ITP patients and in 2 (10%) ITP patients. One of these ITP splenomegaly cases had autoimmune haemolytic anaemia (AIHA), whilst the other patient was under continuous Filgrastim therapy for autoimmune neutropenia, which is also associated with splenomegaly.

No patient with ITP was splenectomised, compared to 5 of 20 (25%) patients with CVID-related ITP. These splenectomies were performed in 1995, 1997, two in 2012, and 2017. The two patients splenectomised in 2012 had a treatment resistant ITP and a significant splenomegaly. The patient splenectomised in 2017 showed a treatment resistant AIHA as well as neutropenia with suspected lymphoma which led to the decision of splenectomy.

Autoimmune manifestations other than ITP or AIHA were present in 3 (15%) patients with ITP and in 7 (35%) patients with CVID-related ITP, as shown in Table 1. Three of these 7 (43%) patients had more than one further autoimmune manifestation, including autoimmune gastritis, type 1 diabetes, autoimmune hepatitis, coeliac disease, seronegative rheumatoid arthritis, sarcoidosis, Hashimoto’s thyroiditis, and optic neuritis (Table S1). Furthermore, one patient was diagnosed with transverse myelitis, which was categorized as an autoimmune manifestation.

### CVID B-cell phenotype and genetics

CVID patients were classified using the EUROclass classification [23]. Fourteen of twenty (70%) patients with CVID-related ITP were classified as B + smB-CD21^low^ (> 1% B-cells, < 2% switched memory B-cells, > 10% CD21^low^ B-cells). This classification, as well as further CVID characteristics, are shown in Table S2.

Genetic testing was performed for 13 of 20 CVID patients. For 3 patients**,** panel sequencing was performed using a panel of immune genes, and for 10 patients, whole exome sequencing was performed. Due to a variety of reasons such as refused consent for genetic testing, personal reasons like moving and the death of one patient, there is missing data of 7 patients. In 13 tested patients with CVID-related ITP, five potentially relevant variants were found (Table 3). The TNFRSF13B variant in patient 6 is, in homozygous state, a known CVID disease mutation [31]. In heterozygous state, as in our patient, the variant is considered a risk factor with low penetrance [32]. The variants identified in PIK3CD, TCF3 and NFKB1 in patients 4, 5 and 7 represent putative dominant disease mutations that have not yet been experimentally tested [33, 34].

**Table 3:** Mutations found in patients with CVID-related ITP

Patient	Gene	Genotype	Protein^a^	dbSNP	Zytogtism	Allele Frequency^b^ (%)	Classification^c^
4	PIK3CD	c.1073T > C	p.Val358Ala	rs188586233	Heterozygous	0.015	III
4	PIK3CD	c.436T > A	p.Phe146Ile	rs142285826	Heterozygous	0.136	II
5	TCF3	c.1896G > C	p.Glu623Asp	rs1198582771	Heterozygous	< 0.001	III
6	TNFRSF13B	c.310T > C	p.Cys104Arg	rs34557412	Heterozygous	0.348	IV
7	NFKB1	c.2831C > A	p.Thr944Asn	rs143882681	Heterozygous	0.032	III

*CVID* Common variable immunodeficiency, *dbSNP* database of single nucleotide polymorphisms, *ITP* immune thrombocytopenia

^a^HGVS nomenclature

^b^gnomAD v2.1.1

^c^According to ACMG guidelines

### Immune phenotype in both groups

An overview of immune parameters measured at presentation in our outpatient clinic is given in Table S3. Blood samples from 18 of 20 patients with ITP and from all 20 CVID-related ITP patients were investigated for immunodeficiencies.

Whilst none of the patients with ITP had a history of immunodeficiency at the point of enrolment, our assessment showed several immunological abnormalities. Of the 18 screened patients, 5 (28%) showed a reduced immunoglobulin level, not fulfilling CVID criteria (Table 4), whilst 7 (39%) were lymphocytopenic.

**Table 4:** Immunodeficiencies in ITP patients

Patient ID	Immunodeficiencies found in our Outpatient clinic
ITP 1	Mild selective IgM deficiency
ITP 2	B-cell lymphocytopenia (40/µl), normal B-cell maturation likely due to steroid therapy
ITP 3	Mild IgG3 subclass deficiency, B-cell lymphocytopenia (50/µl) with normal B-cell maturation
ITP 4^a^	Severe CD4 + T-cell lymphocytopenia (90/µl) and reduced naïve CD4 + T-cells
ITP 5	Unclassified antibody deficiency
ITP 6	Unclassified antibody deficiency
ITP 7	Mild CD-4 + T-cell lymphocytopenia (350/µl) with reduced naïve CD4 + T-cells
ITP 8	IgG3 subclass deficiency
ITP 9^a^	Severe CD4 + (20/µl) and CD8 + T-cell lymphocytopenia, naïve CD4 + T-cells below the lower limit
ITP 10^a^	Severe CD4 + (190/µl) and CD8 + T-cell lymphocytopenia

*ITP* Immune thrombocytopenia; pathological findings in immunological testing in patients with ITP and no further immunodeficiency. Unclassified antibody deficiency as defined in https://esid.org/Working-Parties/Registry-Working-Party/Diagnosis-criteria

^a^Required clinical management and treatment

Of the 5 with reduced immunoglobulin levels, Patient 1 had a mild selective IgM deficiency and Patient 3 showed a mild IgG3 subclass deficiency. Patients 5 and 6 showed an unclassified antibody deficiency as defined by the ESID criteria [35]. One had an IgG- and IgM-deficiency with normal B-cells and memory B-cells, whilst the other one showed IgA-, IgM- and IgG2-, IgG3- and IgG4-deficiencies, as well as fewer memory B-cells, but a monoclonal increase in IgG1. Patient 8 had an IgG3 subclass deficiency.

Of the 7 lymphocytopenic patients, 3 showed a reduction in B-cells but without abnormalities in B-cell maturation whilst 4 patients showed a reduction in CD4 + or CD8 + T-cells as shown in Table 4. In two patients, CD4 + T-cells were severely reduced and in one further patient naïve, CD4 + T-cells were below the lower limit of detection. Therefore, those three patients had to begin treatment, such as regular prophylaxis for pneumocystis jirovecii pneumonia.

**Fig. 2:**
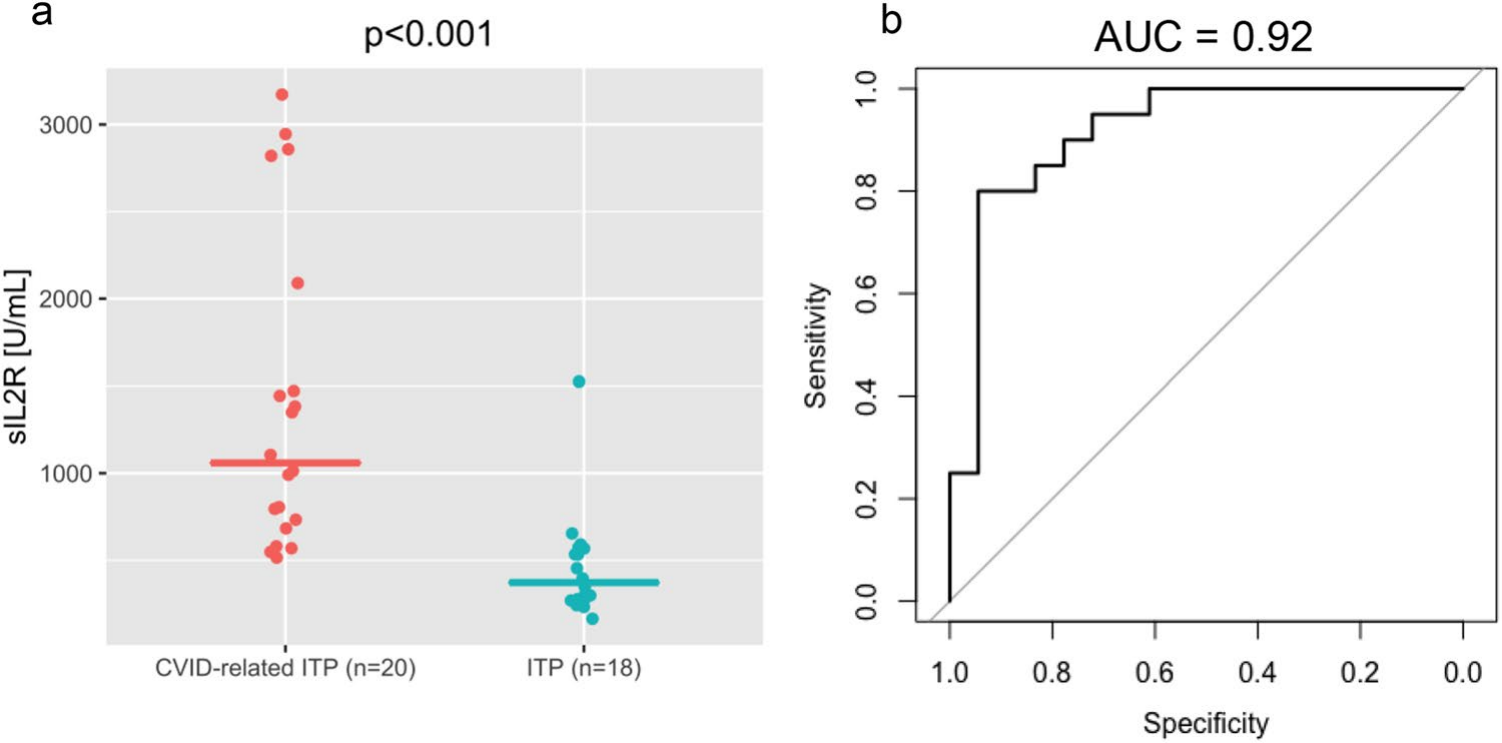
Soluble interleukin 2 receptor differs between patients with CVID-related ITP and ITP. **a**: Distribution of soluble Interleukin-2 Receptor concentrations in patients with CVID-related ITP and ITP patients. **b**: Receiver Operating Characteristic of sIL-2R for discriminating between CVID-related ITP and ITP patients. CVID = Common variable immunodeficiency; ITP = Immune thrombocytopenia; sIL-2R= soluble IL2 receptor. sIL-2R is significantly different (p <0.001) between both groups

Soluble interleukin 2 receptor (sIL-2R) was significantly higher in CVID-related ITP versus ITP patients (Fig. 2, *p* < 0.001). The Area under the Receiver Operating Characteristic for sIL-2R, predicting CVID status amongst all ITP patients and patients with CVID-related ITP was 0.92 (95% confidence interval = 0.82–1.00). The Receiver Operating Curve, as well as the distribution of sIL-2R concentrations in both groups, is shown in Fig. 2.

Figure S1 shows the IgG concentrations of both groups before initiation of immunoglobulin therapy. Patients who were already receiving immunoglobulin therapy at the time of presentation were excluded from this analysis. IgG concentrations differed significantly in both groups (median: patients with CVID-related ITP: 1.96 g/l, ITP: 9.63 g/l). The median relative CD21^low^ B-cells were higher in patients with CVID-related ITP than in ITP patients (14.5 vs. 3.4%, *p* < 0.001) (Table S3). In fact, CD21^low^ B cells in 14 of 17 (82%) ITP patients were within the normal range (0.9–7.6%), whereas they were elevated in 17 of 18 (94%) CVID-related ITP patients. Further, memory B-cells were significantly lower in CVID-related ITP patients (median = 1.1 vs. 16.7%, *p* < 0.01). Platelet counts at initial presentation in the immunodeficiency outpatient clinic showed a median of  176 (127 - 237/nL) in patients with CVID-related ITP and 65/nL (47–96/nL) in patients with ITP (*p* < 0.001) (Table S3, Figure S2). Importantly, at this timepoint, 14 of 19 (74%) CVID-related ITP patients were in remission, whilst 16 of 18 (89%) ITP patients were undergoing ITP therapy.

## Discussion

This study investigates similarities and differences of ITP and CVID-related ITP, using retrospective and prospective analyses for a comprehensive clinical and immunological characterisation of these conditions. Previous research has shown important links between immunodeficiencies and autoimmune cytopenias; [8, 11, 13, 17] however, the pathophysiological and clinical interactions have yet to be fully explored.

A key finding of this study was that although patients with CVID-related ITP had slightly more severe bleeding at initial diagnosis compared to patients with ITP, the clinical course of ITP was significantly milder in patients with CVID-related ITP with respect to the need of treatment, along with higher rates of remission. The milder clinical course in patients with CVID-related ITP may in part be due to the regular IgG substitution dose therapy these patients received, whilst none of the ITP patients received continuous immunoglobulin replacement treatment during the observation period of several years. This hypothesis is supported by the observation that relapses of ITP in patients with CVID-related ITP, occurred more frequently before initiation of IgG substitution therapy, which is in line with findings by Wang et al. and Agarwal et al. [13, 36].

Immunoglobulin replacement therapy in CVID is usually given either intravenously (IVIG) or subcutaneously (SCIG). In our centre, most patients receive their replacement therapy with a dose of 0.4 g/kg body weight/month, given subcutaneously so that they can administer it themselves. Whilst the primary aim of this is to prevent infections, our findings suggest that the continuous immunoglobulin replacement therapy might also have a positive effect on platelet counts in patients with comorbid ITP. Efficacy of IgG replacement in CVID-related ITP has already been shown by previous studies, with not only a good response to low dose immunoglobulins, but also the possibility of reducing steroid treatments [36, 37]. Interestingly a study comparing the efficacy and safety of IVIG and SCIG treatment in CVID-related ITP could show that an IgG trough level under 7 g/L is a key factor for the development of ITP bouts [38].

Currently, only short-term high dose (1–2 g/kg body weight) intravenous immunoglobulins (IVIG) are a standard therapy for ITP patients with severe bleeding complications requiring a rapid increase in platelet counts [39]. However, the effect on platelet counts under high-dose IVIGs remains transient. Continuous IgG therapy in a low dose of 0.4 g/kg body weight may be a therapeutic option for ITP patients without CVID who are refractory to standard therapy, especially if applied subcutaneously, which reduces serum level fluctuations. As of yet, there have been no clinical trials addressing this hypothesis.

Patients with CVID-related ITP were frequently classified as type CD21^low^ with the EUROclass classification [23]. This has already been described in other studies [14, 21, 23, 40] and is currently the most validated marker to screen for predisposition to autoimmune diseases in CVID patients. In our study, interestingly, CD21^low^ B-cells were significantly increased in patients with CVID-related ITP but were mostly within the normal range amongst patients with ITP. So far, there is only very limited data characterising B cell subsets in patients with ITP [41]. In our ITP cohort, B cell subsets were within the normal range.

ITP often precedes the diagnosis of CVID [14, 17, 19, 36, 42]. In our study, the median time from ITP to CVID diagnosis was 7.5 years. Considering this, we suggest screening all ITP patients for CVID by measuring IgG and IgA levels before treatment initiation. In patients receiving high dose IVIG therapy or steroid therapy, this may not always be conclusive as the treatment affects the measured IgG concentrations.

Previous research has found higher concentrations of the inflammatory marker sIL-2R in patients with CVID compared to healthy controls [43–45]. Pathophysiologically elevation in sIL-2R is associated with T-cell activation, which is typical in CVID where T- and B-cell defects are common [43, 45]. Therefore, sIL-2R could be a complementary diagnostic biomarker for CVID sub-classification and prognosis. Van Stigt et al. recently observed increased sIL-2R concentrations in CVID patients with granulomatous disease and showed that it may predict disease progression and response to treatment [46]. Our study showed that sIL-2R was able to distinguish ITP patients from those with CVID-related ITP with high accuracy. Interestingly, amongst the 18 screened patients with ITP, five had decreased IgA and/or IgM concentrations. However, these patients did not fulfil diagnostic criteria for CVID, and all had normal sIL-2R concentrations. This supports our hypothesis for the use of sIL-2R as a complementary diagnostic marker.

However, sIL-2R can be elevated during infections, inflammation, malignant diseases, and other autoimmune diseases, too [43]. ESID criteria, including a poor response to vaccination and other diagnostic markers such as abnormal B-cell maturation, remain the most established diagnostic methodology.

Interestingly, 70% of our CVID-related ITP patients had splenomegaly. Whilst this finding is in line with previous research [7, 10, 13, 42, 47, 48], it is important to consider the splenomegaly as a differential cause of the thrombocytopenia since ITP remains a diagnosis of exclusion [1, 5]. In order to confidently ascribe the diagnosis of ITP to these patients, ruling out other causes of thrombocytopenia, patients were evaluated by experts. Diagnostic criteria included response to ITP-specific treatment, the extent and course of the thrombocytopenia, clinical parameters, and if clinically required, autoantibody screening as well as bone marrow aspiration.

Our study has strengths and limitations. One strength was the extensive data collection, utilizing treatment and clinical data over the course of decades. Additionally, the monocentric nature of this study allows for more accurate comparisons amongst the patients, as all were examined, treated, and evaluated by the same physicians. A limitation is that the ITP patients without CVID were enrolled from a specialized outpatient clinic representing a cohort with high need for therapy.

Our data suggest that the course of thrombocytopenia in patients with CVID-related ITP is milder under IgG substitution, reducing the need for additional ITP specific therapies. Furthermore, we underline the importance of screening ITP patients for CVID and other immunodeficiencies to detect immune abnormalities early. Besides, we also found patients with reduced immunoglobulin levels as well as severe lymphocytopenia in our ITP cohort, of which three patients needed treatment. For the first time, sIL-2R was shown to effectively discriminate between patients with CVID-related ITP and ITP patients.

### Supplementary Information

Below is the link to the electronic supplementary material.

Supplementary file1 (PDF 165 KB)

## Data Availability

The datasets generated during and/or analysed during the current study are available from the corresponding author on reasonable request.
