# Supplementary material for: Clinical and immunological characterisation of patients with common variable immunodeficiency related immune thrombocytopenia
Source: Clin Exp Med. 2023 Sep 5;23(8):5423–32. doi: 10.1007/s10238-023-01166-2 (PMC10725337; doi:10.1007/s10238-023-01166-2)
Supplement: Supplementary file 1 — Supplementary file1 (PDF 165 KB) [file 10238_2023_1166_MOESM1_ESM.pdf]

Supplementary Tables and Figures

Clinical and Immunological Characterization of Patients with Common Variable Immunodeficiency related Immunothrombocytopenia

Clinical and Experimental Medicine

Authors: Nadia Somasundaram[1], Oliver Meyer[2], Carmen Scheibenbogen[1], Leif Gunnar Hanitsch[1], Anna Stittrich[3], Uwe Kölsch[3], Kirsten Wittke[1]

To whom correspondence should be addressed:
Kirsten Wittke, MD
Institute of Medical Immunology
Charité University, Campus Virchow
Augustenburger Platz 1
13353 Berlin, Germany
Kirsten.wittke@charite.de

Table S1: Accompanying Autoimmune Manifestations other than ITP and AIHA

CVID-related ITP	ITP Patients
Autoimmune gastritis	Hashimoto thyroiditis
Hashimoto's thyroiditis	Autoimmune neutropenia
Autoimmune hepatitis	
Celiac disease	
Type 1 diabetes,<br>Seronegative rheumatoid arthritis	
Sarcoidosis	
Optic neuritis<br>Transverse myelitis	

CVID = Common variable immunodeficiency; ITP = Immune thrombocytopenia

Table S2: CVID Characteristics

	CVID-related ITP (n=20)
**EUROclass classification**	
B+smB-21low	14 (70 %)
B+smB+21low	1 (5 %)
B+smB-21norm	1 (5 %)
B+smB-trhi	1 (5 %)
B-	2 (10 %)
B+smB+21norm	0 (0 %)
**Further Characteristics**	
Susceptibility to infection since childhood	8 (40 %)

CVID = Common variable immunodeficiency; ITP = Immune thrombocytopenia
Frequency and column percentages are shown for nominal variables. For variables with missing data, the number of valid cases is shown.

Table S3: Laboratory parameters of CVID-related ITP Patients and ITP Patients

Parameter	Units	CVID-related ITP [median, IQR]	ITP [median, IQR]	*P*-Value
sIL-2R	U/mL	20; 1059 (720 - 1626)	18; 372 (271 - 560)	**<0.001**
IL-8 after Erylysis	pg/mL	18; 127 (79.8 - 206)	18; 159 (127 - 221)	0.293
Platelets	/nL	20; 176 (127 - 235)	18; 65 (47 - 96)	**<0.001**
IgG	g/L	20; 6.35 (2.84 - 8.12)	18; 9.76 (8.2 - 12.6)	**<0.001**
Naive B cells	%*	18; 66 (54.6 - 69.6)	17; 47.1 (43.2 - 72.3)	0.245
Marginal zone B cells	%*	18; 6.8 (5.5 - 17.2)	17; 9.8 (8.2 - 18.6)	0.099
IgM only memory B cells	%*	18; 1.05 (0.677 - 1.8)	17; 2.6 (1.9 - 4.8)	0.002
Switched memory B cells	%*	16; 1.1 (0.425 - 1.55)	17; 16.7 (9.2 - 22.5)	**<0.001**
Transitional B cells	%*	18; 3.85 (1.12 - 6.97)	16; 1.3 (0.4 - 2.1)	0.017
$CD21^{low}$ CD38low B cells	%*	18; 14.5 (12.6 - 28.1)	17; 3.4 (2.5 - 7.3)	**<0.001**
Switched plasmablasts	%*	14; 0.14 (0.1 - 0.237)	17; 0.7 (0.4 - 1.8)	**<0.001**
Granulocytes	/nL	20; 4.78 (3.31 - 5.47)	17; 3.66 (2.92 - 5.91)	0.428
Lymphocytes	/nL	20; 1.31 (0.853 - 1.77)	17; 1.29 (0.98 - 1.46)	0.772
Monocytes	/nL	20; 0.46 (0.31 - 0.71)	17; 0.37 (0.31 - 0.45)	0.352
NK cells	/nL	20; 0.12 (0.055 - 0.17)	16; 0.08 (0.06 - 0.133)	0.444
g/d TZR T cells	/nL	14; 0.03 (0.02 - 0.06)	15; 0.05 (0.0275 - 0.0625)	0.426
a/b TZR T cells	/nL	15; 1.06 (0.655 - 1.28)	16; 0.95 (0.668 - 1.28)	0.874
CD19 B cells	/nL	19; 0.18 (0.088 - 0.29)	17; 0.16 (0.1 - 0.2)	0.512
CD3 T cells	/nL	20; 0.935 (0.68 - 1.27)	17; 1 (0.71 - 1.29)	0.903
CD4 T cells	/nL	20; 0.47 (0.378 - 0.857)	17; 0.63 (0.32 - 0.77)	0.855
CD8 T cells	/nL	20; 0.34 (0.215 - 0.685)	17; 0.35 (0.16 - 0.45)	0.843
Interleukin-2	pg/mL	19; 352 (189 - 464)	17; 210 (144 - 383)	0.254
Mannose-binding lectin	ng/mL	16; 1068 (61.1 - 3288)	16; 1029 (513 - 2199)	0.865
Hemoglobine	g/dL	20; 13.7 (12.9 - 14.3)	18; 14.1 (12.8 - 15.3)	0.342
White Blood Count	/nL	20; 6.65 (4.64 - 8.42)	18; 5.5 (4.35 - 6.67)	0.320
Immunoglobulin A	g/L	20; 0.105 (0.1 - 0.133)	18; 1.45 (1.12 - 1.95)	**<0.001**
Immunoglobulin M	g/L	20; 0.2 (0.128 - 0.355)	18; 0.835 (0.535 - 1.22)	**<0.001**
Immunoglobulin E	g/L	17; 0.2 (0.2 - 0.5)	17; 35.7 (11.5 - 69.4)	**<0.001**
Free lambda light chains	mg/L	16; 7.04 (4.84 - 8.79)	17; 13.3 (11.2 - 16.3)	**<0.001**
Free kappa light chains	mg/L	16; 4.53 (1.07 - 6.29)	17; 14.3 (11.7 - 18.1)	**<0.001**
Free kappa/Free lambda ratio	mg/L	16; 0.775 (0.575 - 1.01)	17; 1.22 (1.02 - 1.33)	0.006
IgG1	g/L	20; 3.67 (1.86 – 4.39)	18; 5.21 (4.75 - 6.05)	0.806
IgG2	g/L	20; 1.99 (0.476 – 2.68)	18; 3.03 (2.33 - 4.53)	0.942
IgG3	g/L	20; 0.37 (0.285 - 0.601)	18; 0.46 (0.29 - 0.59)	0.654
IgG4	g/L	17; 0.04 (0.0058 - 0.11)	18; 0.253 (0.11 - 0.306)	**<0.001**
C-Reactive Protein (CRP)	mg/L	19; 2.5 (1.3 - 5.45)	18; 0.65 (0.6 - 1.23)	0.011
Lactate Dehydrogenase (LDH)	U/L	18; 215 (197 - 243)	18; 249 (201 - 300)	0.145

CVID = Common variable immunodeficiency; ITP = Immune thrombocytopenia
Median and interquartile ranges (IQR) are shown for continuous variables. The number of cases with valid data is shown before the semi-colon. * per cent of total CD19 B cells

Fig. S1 IgG Patients with CVID-related ITP and ITP before immunoglobin substitution therapy

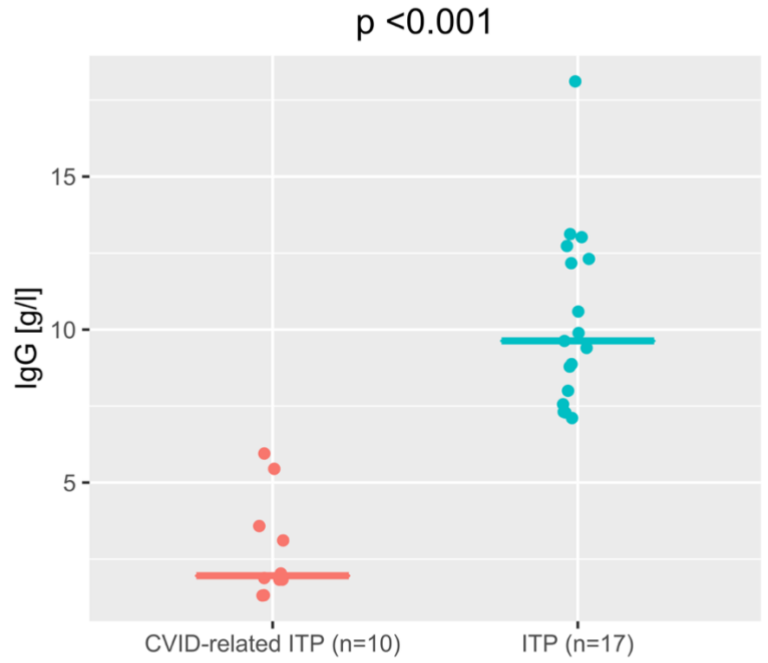

CVID = Common variable immunodeficiency; ITP = Immune thrombocytopenia
IgG is significantly different between all three groups. One ITP patient was not under immunoglobulin substitution therapy but under high-dose immunoglobulin therapy

Fig. S2 Platelets in Patients with CVID-related ITP and ITP

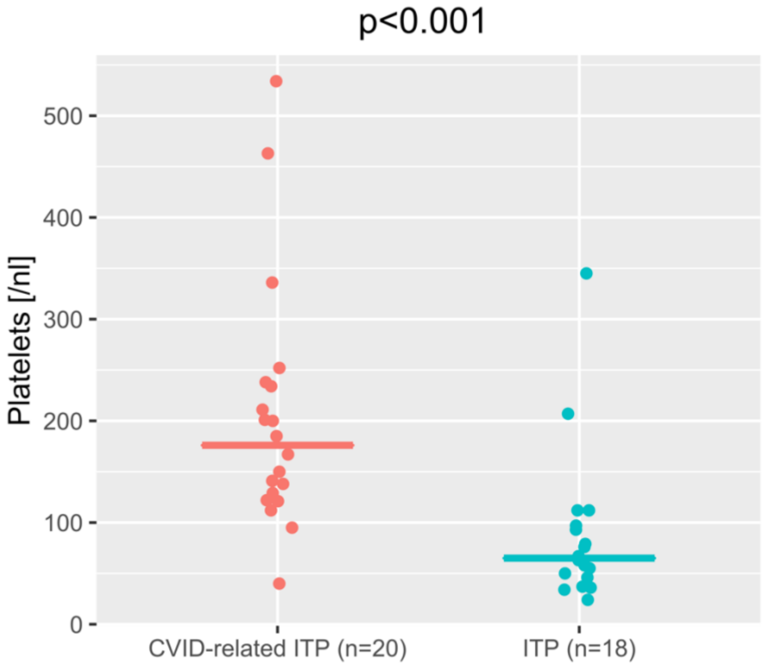

CVID = Common variable immunodeficiency; ITP = Immune thrombocytopenia
Platelets are significantly different ($p<0.001$) between both groups
